# Intrinsic Fatigue Limit and the Minimum Fatigue Crack Growth Threshold

**DOI:** 10.3390/ma16175874

**Published:** 2023-08-28

**Authors:** Mirco D. Chapetti, Nenad Gubeljak, Dražan Kozak

**Affiliations:** 1 Laboratory of Experimental Mechanics, INTEMA, National University of Mar del Plata—CONICET, Av. Colón 10.850, Mar del Plata 7600, Argentina; 2 Faculty of Mechanical Engineering, University of Maribor, Smetanova ul. 17, 2000 Maribor, Slovenia; 3 Mechanical Engineering Faculty, University of Slavonski Brod, Trg Ivane Brlic-Mazuranic 2, HR-35000 Slavonski Brod, Croatia

**Keywords:** intrinsic fatigue limit, microstructural fatigue threshold, material defects, fracture mechanics

## Abstract

In the field of long-life fatigue, predicting fatigue lives and limits for mechanical components is crucial for ensuring reliability and safety. Fracture mechanics tools have enabled the estimation of fatigue lives for components with small cracks or defects. However, when dealing with defects larger than the microstructural characteristic size, estimating the fatigue resistance of a material requires determining the cyclic resistance curve for the defect-free matrix, which depends on knowledge of the material’s intrinsic fatigue limit. This study focuses on the experimental evidence regarding the intrinsic fatigue limit and its correlation with naturally nucleated non-propagating cracks. Fracture mechanics models for small crack propagation are introduced, and their disparities and limitations are analyzed. The concept of intrinsic fatigue limit is then introduced and applied to reanalyze a recent publication. Methods for estimating the intrinsic fatigue limit are explored and applied to experimental results reported in the literature. The need to clarify and accurately predict the intrinsic fatigue limit is highlighted in alloys where the processing generates defects larger than the microstructural size of the matrix, as often observed in materials and components produced using additive manufacturing.

## 1. Introduction

In the field of long-life fatigue, prediction approaches are gaining significant attention from researchers [1,2,3,4]. This is primarily due to the increased demand for reliability and safety in the design of mechanical components used in industrial applications. With the evolution of fracture mechanics tools, it has become possible to estimate the behavior of short cracks that are less than a millimeter long. This has made it possible to estimate the fatigue lives and limits (or endurance) associated with mechanical components that have small cracks or crack-like defects generated during manufacturing.

In some manufacturing processes, such as additive manufacturing, inherent defects can occur, which eliminate or minimize the initiation stage of fatigue cracks. As a result, it is plausible to assume that the damage process primarily involves the propagation of a crack from the critical defect until it causes the component to fracture. Therefore, fracture mechanics is crucial in developing predictive models [5,6]. This, combined with the advancements in understanding the behavior of small cracks and the development of models that can predict it, has made the damage tolerance methodology the best tool for designing safe mechanical components [1,2,3,4]. It also reduces the need for excessive safety factors, enabling more reliable design and life estimation.

However, fracture mechanics analysis necessitates estimating the resistance curve of the material in the absence of defects [1,2,3,4], whether in terms of the stress range or the range of the stress intensity factor as a function of the crack length: Δσ_th_ vs. *a* or ΔK_th_ vs. *a*. The initial size associated with the damage process is subsequently determined using the maximum expected defect size in the manufactured component. However, if these defects are inherent to the manufacturing process, estimating the resistance curve requires knowledge of the intrinsic fatigue limit of the material, which represents the material’s resistance without defects.

In this study, we begin by recalling fundamental concepts associated with high cycle fatigue damage, which serve as a foundation for analyzing fracture mechanics models, including their assumptions and limitations. Specifically, we focus on the Murakami and Endo [7] and the Chapetti [8] models and those based on the crack closure phenomena [9,10]. Subsequently, we present experimental evidence concerning the fatigue limit and its correlation with the presence of naturally nucleated, non-propagating cracks. This enables us to perceive the limit as a threshold for microcrack propagation. Additionally, fracture mechanics models that estimate the propagation threshold for small cracks are introduced, and comparative analyses to highlight their disparities and limitations are conducted. Furthermore, a recent publication is reanalyzed using the proposed concept of intrinsic fatigue resistance. Finally, we explore the potential methods for estimating the intrinsic material properties necessary for the application of these models. By doing so, we aim to enhance our understanding of fatigue damage mechanisms and pave the way for better assessments of material fatigue behavior.

## 2. High Cycle Fatigue Resistance

**Figure 1:**
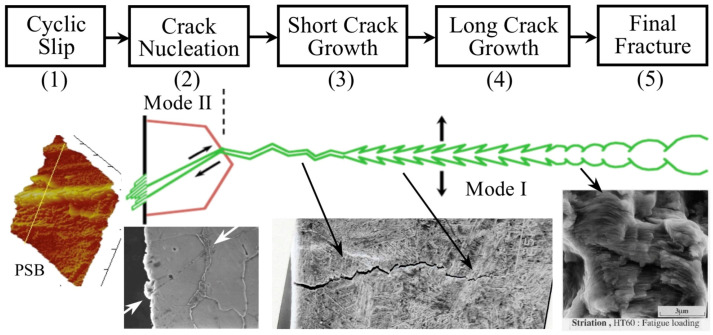
Five stages of mechanical high cycle fatigue damage in metallic materials without defects.

Figure 1 presents a schematic representation of the evolution of a fatigue crack initiated from a polished surface of a defect-free metallic alloy. Additionally, the figure showcases various crack lengths and illustrates the generation of surface damage on carbon steels.

Due to low applied nominal stresses (high cycle fatigue) and surface concentration effects, plastic deformation is confined to specific grains with favorable orientations, minimizing restrictions from neighboring grains. With increasing cycles, damage grows, causing persistent slip bands (PSB) induced by shear stresses, resulting in intrusions and extrusions (stage 1). Localized damage areas lead to microcracks similar in size to the microstructure (e.g., Figure 1, mode II crack arrested at a grain boundary, stage 2). Early microcrack propagation culminates in macrocrack formation (stage 3), and subsequent engineering crack propagation leads to final failure or fracture (stages 4 and 5).

Accurately predicting total fatigue life, encompassing both long fatigue crack propagation and short crack regimes, hinges on properly quantifying fatigue crack initiation life (stages 1 and 2). However, in scenarios involving components with small cracks or crack-like defects, such as additive manufactured metals, slip damage localization and microcrack generation stages may be negligible. Consequently, fatigue behavior becomes dominated by the propagation of undetectable microcracks until component failure. To address this, reliable prediction models for non-detectable cracks (short cracks) are essential [3,7,8,9,10,11,12]. In the subsequent sections, we analyze various aspects of fracture mechanics models utilized for these tasks.

### 2.1. Crack Initiation and Intrinsic Fatigue Limit

The crack initiation mechanism has been a significant focus of research for decades [13,14,15], with numerous models proposed, primarily based on dislocation movement [14,15,16]. Historically, the interpretation of the fatigue limit as the “limit of crack initiation under cyclic stress” was prevalent [17,18,19] (as it is pointed out by Murakami [4]). For cases where a fatigue limit cannot be determined, an “endurance limit” for a specific fatigue life (e.g., 10^7^ cycles) may be considered instead.

**Figure 2:**
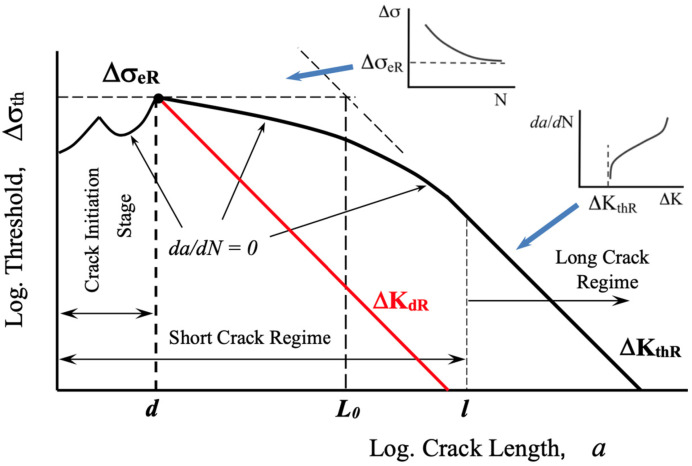
Kitagawa–Takahashi diagram showing the threshold for fatigue crack growth in terms of stress range.

In the present understanding, the high-cycle intrinsic fatigue limit is no longer viewed as a critical stress for crack initiation. Instead, it represents the stress level below which an already initiated micro-crack cannot propagate. In essence, the intrinsic fatigue limit acts as a threshold stress for micro-crack growth. This concept was clearly reported by Miller in reference [6], employing a Kitagawa–Takahashi diagram [20], which illustrates the threshold stress range for crack propagation as a function of crack length (see Figure 2). This limit is material-based and depends on the microstructural characteristic dimension (*d*) [6]. Early evidence supporting this concept can also be found in the thorough analyses of Tanaka et al. [21] and Tokaji et al. [22]. Previous research by Kitano, Chapetti, and their colleagues [23,24,25,26,27,28,29] delved into the position and effective resistance of microstructural barriers, establishing their relation to the fatigue limit. These studies provide additional evidence that the intrinsic fatigue limit for plain and blunt-notched samples is defined by the strongest microstructural barrier. This shift in perspective has brought greater clarity to the fatigue phenomenon, emphasizing the significance of limiting crack growth rather than merely preventing crack initiation. The Kitagawa–Takahashi diagram has become an invaluable tool in fatigue research, aiding in the prediction of crack propagation behavior and enabling more precise and efficient fatigue life assessments in various materials and engineering applications.

**Figure 3:**
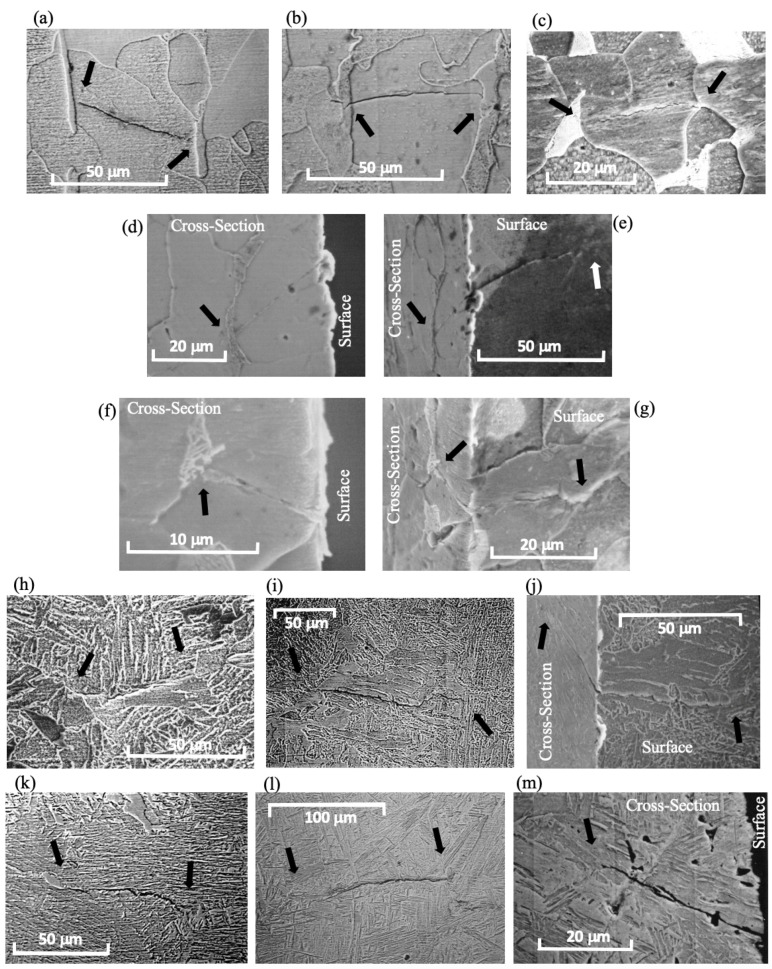
Examples of microstructurally small non-propagating cracks. Ferrite microstructure: (**a**–**c**) [23], (**d**) [28], (**e**–**g**). Ferrite-Bainite microstructure: (**h**) [23,24], (**i**) [24] and (**j**). Bainite-Martensite microstructure: (**k**) [24], (**l**) [28] and (**m**). Photographs (**d**–**g**,**j**,**m**) correspond to cross-sectioning and reveal the internal configuration of the arrested cracks as well as the microstructural barriers. Black and white arrows indicate the location of the tips of non-propagating cracks associated with the intrinsic fatigue limit.

In reference [23,24], three different steel microstructures were analyzed under tension-compression (*R* = −1): ferrite (JIS SS-400, σ_U_ = 438 MPa, H_V_ = 127 Kgf/mm^2^, *d* = 38 μm and Δσ_eR_ = 420 MPa), ferrite-bainite (JIS SW12-3, σ_U_ = 552 MPa, H_V_ = 181 Kgf/mm^2^, *d* = 50 μm and Δσ_eR_ = 510 MPa) and bainite-martensite (JIS SW12-5, σ_U_ = 740 MPa, H_V_ = 288 Kgf/mm^2^, *d* = 50 μm and Δσ_eR_ = 580 MPa). The sample surfaces were totally polished and revealed before testing. Figure 3 illustrates non-propagating cracks found in samples tested at stress levels at or just below the fatigue limit (run-out results for 10^7^ cycles). Some of these cracks are newly published, while others were previously reported in publications [23,24,27,28]. All non-propagating cracks were effectively arrested by the first and strongest barrier directly associated with the material’s intrinsic fatigue limit. These cracks occur naturally without the introduction of artificial defects to localize their nucleation, as is commonly done. It should be noted that naturally nucleated cracks manifest themselves in the weakest configuration along the entire surface of the sample, making their localization arduous and time-consuming. However, this approach represents the most appropriate method for studying crack nucleation processes in any material. Another crucial aspect to consider is the necessity of observing and analyzing not only the surface path of the crack but also, primarily, its depth and the position of the crack tip in relation to microstructural barriers. Figure 3 provides surface observations of the non-propagating (or arrested) cracks associated with the intrinsic fatigue limit of the three microstructures. Additionally, cross-section examples of these cracks clearly demonstrate the influence of microstructural barriers that define the intrinsic fatigue limit and allow for their identification. These barriers represent a microstructural threshold for micro-crack growth. Nowadays, advanced equipment such as Scanning Electron Microscopy-Focused Ion Beam (SEM-FIB) is available, making the task of understanding crack nucleation processes much simpler and faster. Therefore, carrying out this task no longer requires significant effort and should be widely adopted when analyzing these topics.

In reference to [27], Chapetti et al. conducted a comprehensive analysis of the impact of four distinct static strengthening methods on fatigue crack initiation, fatigue limit, and blunt-notch sensitivity in low-carbon steels with a ferrite–pearlite microstructure. The microstructural barriers’ average distance (*d*) was kept constant by maintaining a consistent average grain size, while the effective resistance to crack growth was increased using static strengthening. All microstructures exhibited a ferrite–pearlite configuration with a similar grain size (55 μm). To investigate the intrinsic fatigue limits of each configuration, test specimens were examined at nominal stress levels just below and very close to the experimental intrinsic fatigue limit (similar to the approach used in references [23,24]). In all cases, the non-propagating cracks associated with the intrinsic fatigue limit were consistently found to be arrested by the first grain boundary, with a depth equivalent to the average grain size. These findings provided further compelling evidence that, in most instances, the intrinsic fatigue limit is determined using the strongest microstructural barrier, as previously suggested by Miller [6] (see Figure 2). The resistance to crack propagation is generally greater than the resistance to crack nucleation, reinforcing the critical role of microstructural barriers in defining the intrinsic fatigue limit of the material.

Based on the experimental evidence, the intrinsic fatigue limit can be precisely defined as the capacity of the strongest microstructural barrier (e.g., grain boundary) to arrest a micro-crack. Utilizing the intrinsic fatigue limit, Δσ_eR_, and the average distance from the surface to the strongest microstructural barrier (corresponding to the average microstructural size, *d*), a minimum intrinsic resistance to micro-crack growth (microstructural threshold, ΔK_dR_) can be established for a given load ratio *R*. This formulation was proposed by Chapetti [8] and is depicted in Figure 2, as follows:

(1) $$\Delta K_{\mathrm{dR}}=Y\;\Delta \sigma_{\mathrm{eR}}\;\sqrt{\pi \;d\;}$$

*Y* is the geometric correction factor that is taken conservatively as 0.65 because, in most cases, microstructural short cracks nucleated at surfaces are considered semicircular [23,24,30]. The ‘*R*’ subscript indicates that as Δσ_eR_ is *R*-ratio (load ratio) dependent, ΔK_dR_ also is.

The ΔK_dR_ parameter, a microstructural threshold, represents the minimum driving force we can apply to propagate a crack of size *d*. Furthermore, it defines a minimum intrinsic stress range as a function of crack length associated with the total threshold for crack growth, as shown by the red line in Figure 2.

It is important to mention here that to experimentally analyze this intrinsic microstructural propagation threshold associated with the intrinsic fatigue limit of the material, it is necessary to analyze naturally nucleated cracks. Artificial defects cannot be used because the intrinsic fatigue limit is given by the weakest microstructural configurations in microstructural entities favorably oriented to induce surface strain concentrations. Any artificially introduced defect would neglect this concept and would introduce substantial changes that would make the configuration finally studied different from the one that would naturally be generated when a fatigue crack nucleates.

### 2.2. The Microstructural Threshold ΔK_dR_ and the Surface Strain Concentration

Once the la crack is nucleated and overcome the barrier associated with the intrinsic fatigue limit, several transition processes take place. These include the effect of surface strain concentration, which involves the first 3 or 4 microstructural entities, the transition of the crack opening mode, shifting from mode II during crack nucleation to opening mode I as the crack propagates and develops further, and the development of the crack closure phenomenon (initially null when the crack nucleates). The analysis of crack closure and its fundamental concepts will not be discussed in detail here, but they can be explored in the extensive available literature [31,32,33,34,35,36]. Additionally, the concepts related to the transition of the propagation mode, which depends on various material properties and its microstructure, will not be covered in this discussion. What is important to highlight is the concept of surface strain concentration since it is generally unknown or dismissed by many analyses that only contemplate the development of crack closure as the only phenomenon associated with the development of the propagation threshold, ΔK_th_ (see, for instance [1,2,9,10]). As will be discussed later, this can be the case once the crack has passed 3 or 4 microstructural entities. However, the disregarded phenomenon does not allow extrapolating the effective thresholds of long cracks (ΔK_th,eff_) to microcracks associated with the intrinsic fatigue limit of sizes similar to the microstructural size *d*.

**Figure 4:**
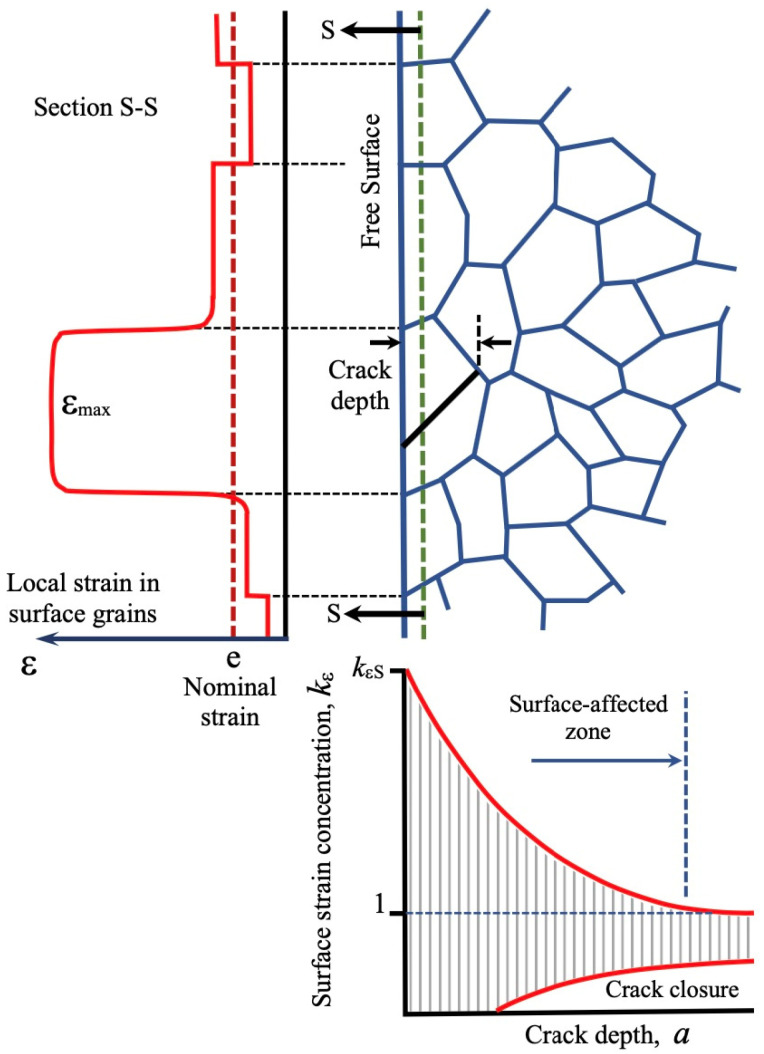
Surface strain redistribution. After Abdel-Raouf et al. [37,38].

Figure 4 illustrates the concept proposed by Abdel-Raouf, Topper, and Plumtree [37,38], elucidating the inherent surface strain concentration phenomenon. The explanation centers around the disparity in deformation between the material at the free surface and the interior, where neighboring grains provide support (with increasing constraint at depth). Additionally, the free surface of polycrystalline alloys comprises numerous randomly oriented grains, each with different slip system orientations relative to the loading axis. This leads to a strain redistribution process. The strain in the surface is accommodated due to the lack of constraint, and the local strain is proportionate to the corresponding grain orientation. Favorably oriented grains undergo more surface deformation and experience a higher level of localized slip, thus serving as preferred sites for crack initiation.

In contrast, within the material’s interior, grains mutually support each other, leading to a decrease in local strain with depth into the specimen until it approaches the nominal strain range. This results in a constraint gradient, which arises from the variation in grain orientations and the lack of constraint at the surface. As the local strain diminishes with depth, it approaches the nominal strain range due to increased constraint and strain compatibility requirements. Furthermore, they argued that the rate of decay is influenced by the average grain size *d*, meaning that larger grains exhibit deeper local resolved shear strain (greater deformation) and have less surface area per unit volume of contact with neighboring grains, consequently experiencing less constraint.

After the crack departs from the region influenced by these phenomena, the dominant factor in the development of the crack growth threshold, ΔK_th_, becomes the crack closure effect, which initiates during the early stages of crack growth.

Those phenomena were considered by Chapetti when proposing his model. The minimum threshold ΔK_dR_ (see Equation (1)) is defined by the experimentally measured intrinsic fatigue limit, Δσ_eR_, which implicitly includes and quantifies all the phenomena mentioned above. Additional work on the estimation of the complementary extension force due to the surface strain concentration is reported in reference [26].

The concentration of deformations on the surface plays a critical role in defining the intrinsic fatigue limit because it is dependent on the weakest configuration that generates the dominant crack. This further supports the difficulty in extrapolating the effective threshold for long cracks, ΔK_th,eff_ (threshold without crack closure), to determine a non-propagating crack length connected to the intrinsic fatigue limit. The value obtained from this method cannot be linked to the microstructure and does not represent the length physically associated with the intrinsic fatigue limit of the material.

**Figure 5:**
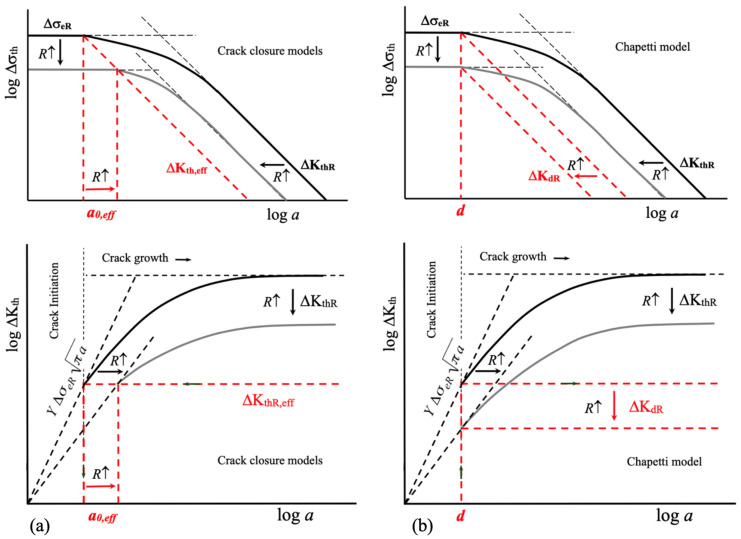
(**a**) Chapetti model. (**b**) Crack closure models.

Figure 5 shows the basic differences between models that use the concept of crack closure and the Chapetti model. The underlying assumptions associated with the intrinsic fatigue limit are different. This is important in order to correctly define an intrinsic fatigue limit and the associated variables. While the former assumes that the intrinsic fatigue limit is determined by the effective crack propagation threshold, ΔK_th,eff_ (thus, the non-propagating crack length *a*_0,eff_ associated with the intrinsic fatigue limit increases as the load ratio increases because Δσ_eR_ decreases), the latter defines the microstructural threshold ΔK_dR_ considering a fixed non-propagating crack length given by the average microstructural size *d*, yielding different values of ΔK_dR_ for different fatigue limits Δσ_eR_ (different load ratio *R*).

Table 1 presents ΔK_dR_ and *a*_0,eff_ results for values of Δσ_eR_, ΔK_th_, and *d* obtained from the literature for various metals [21,27,29,39,40,41,42], along with reported or estimated values of the effective threshold using the expression ΔK_th,eff_ ≈ 1.3 × 10^−5^ E [3,43], and E = 200 GPa.

It is evident that the crack closure model hypothesis gives rise to non-propagating cracks linked to the intrinsic fatigue limit that can extend up to four times the size of the microstructure. In the case of materials with small grain sizes, this effect can be even more pronounced. For instance, in the case of S20C steel with a diameter of 7.8 μm and a stress ratio *R* = 0, the estimated non-propagating crack size can reach up to twelve times the microstructural size.

*a*_0,eff_ is, by definition and as a result of the crack closure model hypothesis, directly independent of *d* and is only defined, for a given ΔK_th,eff_, by the intrinsic fatigue limit Δσ_eR_. However, it is possible to increase the intrinsic fatigue limit without varying the microstructural size *d*. This can be observed, for example, in the previously referenced work [27], where five ferritic steels with similar microstructural sizes (average ferrite grain size *d* = 55 μm) but different intrinsic fatigue limits generated using the addition of alloying elements or precipitation heat treatment are analyzed.

If we analyze the case of the high-strength steel JIS SUJ2 [40], *a*_0,eff_ is estimated to be 1 μm, one-tenth of the microstructural size *d* (10 μm). This creates a configuration that requires an appropriate phenomenological explanation to be justified, which does not allow the use of the entire microstructural entity to explain crack nucleation.

On the other hand, in the case of ultrafine-grained steel (0.8 μm), the concept of crack closure applied to the intrinsic fatigue limit yields an *a*_0,eff_ ten times the microstructural size *d*. Experimental evidence shows that it was not possible to find non-propagating cracks larger than *d* associated with the intrinsic fatigue limit. This was only possible for fatigue limits with stress concentrators: *a*_np_ = 2 μm for *k*_t_ = 2, and *a*_np_ = 4 μm for *k*_t_ = 2.8 (see details in reference [29]), far from the estimated *a*_0,eff_ = 10*d*.

This author has not been able to find conclusive experimental evidence in the literature correlating the observed non-propagating crack length at the intrinsic fatigue limit for a given load ratio and the length estimated using the ΔK_th,eff_ criterion. In fact, this author proposed Equation (1) as a minimum threshold for microcrack growth for the intrinsic fatigue limit as a result of being unable to explain the experimental observations using only the concept of crack closure [24,26,28].

### 2.3. Fatigue Crack Propagation Threshold Curve: ΔK_th_ vs. a, estimation Models

Let us analyze the transition between the threshold associated with the intrinsic fatigue limit, Δσ_eR_ (or ΔK_dR_, the minimum threshold value), and the threshold for long crack propagation, ΔK_thR_ (the maximum threshold value). This transition is complex as it involves dealing with very small cracks in threshold configurations within a specific range of crack lengths. The Chapetti model [8] and the Murakami–Endo model [7] are used here to analyze the short crack regime. Refer to references [11,44] for a detailed analysis of this range of short cracks and a discussion of hypotheses regarding various prediction models for threshold curves and their differences.

Based on the analysis conducted in the previous section, models that rely solely on the concept of crack closure will not be used. Neither is the El Haddad model [12], as it does not allow for the definition of a minimum threshold value in terms of ΔK_th_ associated with the intrinsic fatigue limit (in fact, this model indicates ΔK_th_ = 0 for *a* = 0).

#### 2.3.1. The Chapetti Model

In Section 2.1, Chapetti’s hypothesis was introduced, stating that the minimum ΔK_th_ associated with the intrinsic fatigue limit is equal to ΔK_dR_. It can be estimated using the same intrinsic fatigue limit and the average microstructural size *d* (e.g., grain size) as expressed in Equation (1) (refer to Figure 2). This hypothesis is based on the idea that the intrinsic fatigue limit is determined using the capacity of the strongest microstructural barrier (e.g., grain boundary) to arrest a micro-crack. The parameter ΔK_dR_ represents the minimum driving force required to propagate a crack of size *d*. This value serves as the starting point for the threshold, which then evolves until it reaches its maximum value, defined by the long crack threshold, ΔK_thR_.

According to Chapetti’s model, the threshold for crack growth comprises both a “microstructural” component, ΔK_dR_, and an “extrinsic” component, which is a function of crack length and is equal to (ΔK_th_ − ΔK_dR_). As the crack length increases, this extrinsic component fully develops and reaches a maximum value (ΔK_thR_ − ΔK_dR_) for long cracks. Chapetti proposed an exponential function to model the development of (ΔK_th_ − ΔK_dR_), leading to the following expression for estimating the threshold for short crack growth as a function of crack length [8]:

(2) $$\Delta K_{\mathrm{th}}=\Delta K_{\mathrm{dR}}+\left(\Delta K_{\mathrm{thR}}-\Delta K_{\mathrm{dR}}\right)\;\left[1-e^{-k\left(a-d\right)}\right]\;\;\;\;\;a\geq d$$

where *a* is the crack length and *k* is a material constant given by the following expression [8]:

(3) $$k=\frac{1}{4\;d}\;\frac{\Delta K_{\mathrm{dR}}}{\left(\Delta K_{\mathrm{thR}}-\Delta K_{\mathrm{dR}}\right)}$$

Equations (2) and (3) are fully defined once Δσ_eR_, ΔK_thR_, and *d* are known. Further details of this model can be found in references [8,11].

In Chapetti’s model, the resistance curve ΔK_th_ vs. *a* does not require a “fitting parameter” *k*. Equation (3) is derived from hypotheses associated with the proposed model, and factor 4 is the only contribution stemming from both the model’s hypotheses and a fitting procedure applied to various sets of threshold data for short cracks at the time of proposal [8]. However, when utilizing the model, Equations (2) and (3) do not necessitate any fitting procedure and offer a direct estimation of the threshold curve, providing a reliable and pure prediction of crack growth thresholds.

Equations (2) and (3) are widely employed by various authors to estimate the resistance curve in models considering that the minimum crack propagation threshold, ΔK_th_, associated with the intrinsic fatigue limit, is represented by the effective propagation threshold, ΔK_th,eff_ (crack closure models). This approach yields acceptable results, particularly in analyses where the intrinsic length *a*_0,eff_ related to the fatigue limit exhibits dimensions similar to the microstructural size *d*, but it cannot be generalized, as this practice can yield considerably different results, as we have seen in Section 2.2.

For more details on the prediction models that use the crack closure concept, references [9] and [10] can be consulted for the proposals of McEvily et al. and Tanaka and Akiniwa, respectively. McEvily’s model is often confused with Chapetti’s model, but they only have in common the exponential mathematical expression to express the development of the propagation threshold since the models are based on substantially different hypotheses. Some studies even use the McEvily model (and its hypotheses) but resort to the parameter *k* of the Chapetti model (Equation (3)) to estimate its exponential development parameter (see, for instance, [45,46]). These procedures mix hypotheses and run the risk of generating weakly supported conclusions, mainly for alloys with relatively small defects.

#### 2.3.2. The Murakami–Endo Model

The Murakami–Endo model [7] provides an estimation of the threshold ΔK_th_ for small cracks and defects by considering the Vickers hardness, H_V_, and the *area*^1/2^ parameter, which is the square root of the area resulting from projecting a small defect or crack onto a plane perpendicular to the maximum principal stress. To estimate the threshold stress range Δσ_th_ for surface cracks with *R* = −1 [4,7], Murakami and Endo proposed the following expression:

(4) Δσth=2.86  HV+120area16 

where *area*^1/2^ is in μm and *H_V_* in kgf/mm^2^, given Δ*σ_th_* in MPa. In the case of the threshold for short crack growth, in terms of the ΔK, the following expression was proposed [4,7]:

(5) ΔKth=0.0033 HV+120 area13

Equations (4) and (5) are applicable under fully reversed loading conditions, i.e., for load ratio *R* = −1. The effect of load ratio is taken into account by the model by multiplying the expressions (4) and (5) by the following factor [4,7]:

(6) $${\left(\frac{1-R}{2}\right)}^{\alpha }$$

with α given by the expression α = 0.266 + *H_V_* × 10^−4^.

An essential feature of this model is its proposition of a constant potential relationship between the propagation threshold and the defect size. However, as ΔK_th_ approaches the long crack growth threshold, it no longer depends on the defect size, as we have exited the domain of short cracks. The threshold for long crack growth thus establishes an upper limit (in terms of crack length) for the applicability of the Murakami–Endo model, as demonstrated in reference [47].

## 3. The Intrinsic Fatigue Limit

Here, we will delve into the importance of distinguishing between the intrinsic fatigue limit of the material, given by Δσ_eR_ or ΔK_dR_, and the fatigue limit of a material containing small cracks or defects larger than the microstructural size *d*. In these materials, what is being experimentally measured or estimated using the Murakami model is the fatigue limit of the matrix-defect ensemble. In this case, it is not possible to experimentally measure the intrinsic fatigue limit of the matrix, so it becomes necessary to estimate it appropriately. Next, it will be demonstrated how this intrinsic fatigue limit represents a lower bound to the Murakami model and how it could be estimated to properly apply fracture mechanics models in materials with defects larger than *d*, which are, in many cases, inherent to the manufacturing process.

**Figure 6:**
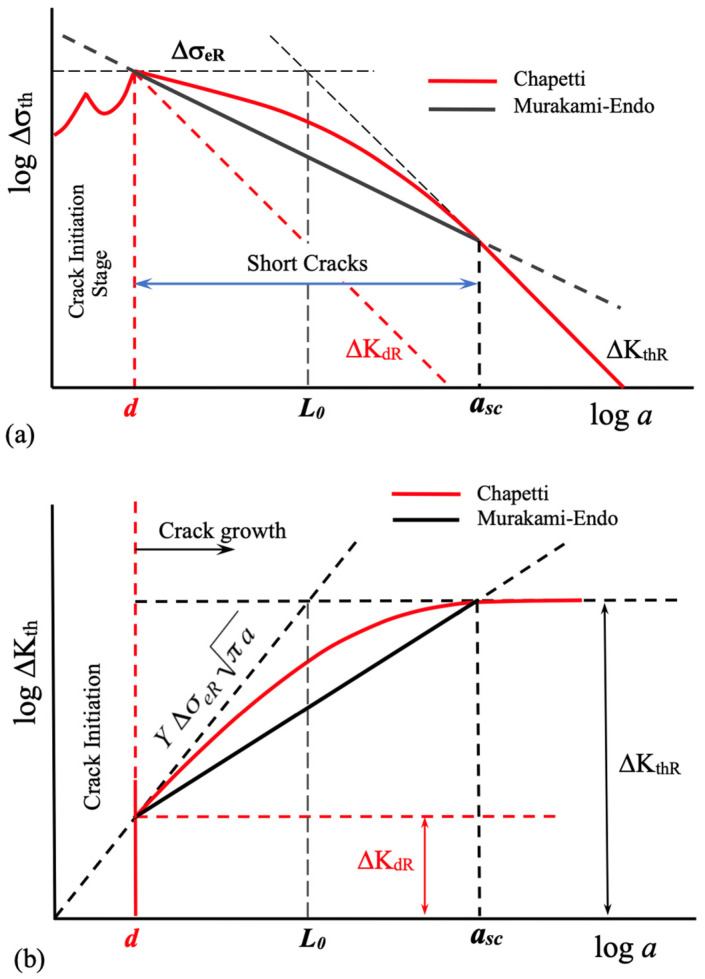
Schematic representation of the estimated threshold for crack growth. Murakami–Endo model (dark line) and Chapetti model (red line). (**a**) Kitagawa–Takahashi type diagram, Δσ_th_ vs. *a*. (**b**) Threshold in terms of the stress intensity factor range, ΔK_th_ vs. *a*. Limits of the Murakami–Endo model are shown in terms of the crack length: *d*, minimum limit given by the fatigue limit (Δσ_eR_ or ΔK_dR_), and *a*_sc_, maximum limit given by the threshold for log cracks, ΔK_thR_.

Figure 6 illustrates the propagation thresholds in terms of Δσ_th_ (Figure 6a, K-T diagram) or ΔK_th_ (Figure 6b), as predicted by the Murakami–Endo and Chapetti models. These models provide insights into the critical values required for crack propagation. The Murakami model is bounded by both lower and upper limits. The upper limit corresponds to the long crack threshold value, ΔK_thR_, discussed and exemplified in reference [47]. On the other hand, the lower limit is determined by the microstructural size *d* associated with the material’s intrinsic fatigue limit. As reviewed by Miller [6,30], any defect or initial crack smaller than this *d* value would have no significant impact on the intrinsic fatigue limit of the material.

In the schematic representation of Figure 6, the predictions of the Murakami–Endo and Chapetti models are matched when *a* = *d*. This assumption implies that both models predict the same intrinsic fatigue limit of the material when there are no defects or cracks exceeding the size of *d*. In fact, this is a hypothesis that has been applied when conducting analyses using various prediction models, as in the works of Schönbauer and Mayer [48]. In the remaining part of this section and in the following ones, this hypothesis is examined in more detail, particularly when utilizing it to estimate ΔK_dR_. Let us remember that in the case of the Chapetti model, Δσ_eR_ is a data (input) that needs to be measured or estimated, while in the Murakami–Endo model, Δσ_e_ (fatigue limit) is estimated by the model itself based on the hardness and size of the defect or crack.

The lower limit of the Murakami–Endo model has not been adequately addressed in previous analyses, as discussed in references [11]. Some claims have even suggested that the intrinsic fatigue limit estimated by the Murakami–Endo model could involve up to 3 or 4 microstructural entities [4,49,50]. This implies that defects or cracks smaller than 3*d* or 4*d* would not have an impact on the fatigue limit of the material or component. However, this author believes that such claims arise from the analysis of works involving artificial defects or metals with very low strength relative to other materials. In the last case, the fatigue strength is significantly low in terms of ΔK_th_ compared to the threshold of long cracks, ΔK_thR_, which is associated with a wider range of short cracks. Similar observations can be made when analyzing relatively low-strength copper alloys, as demonstrated in reference [42], where the intrinsic fatigue limit is associated with 2*d*. Furthermore, the short crack range (*d*-*a*_sc_ in Figure 2) increases as the strength or hardness of the metal decreases, as shown and explained in reference [11], for instance. This leads to a situation where the evolution of the fatigue limit with the crack length (or defect size) exhibits a small average slope in the Kitagawa–Takahashi diagram (see Figure 1). Consequently, it raises the likelihood that the material’s intrinsic fatigue limit is linked to micro-crack sizes that surpass the microstructural size, *d*. For further examination of this matter, reference [50] can also be analyzed.

In order to illustrate the challenges associated with defining intrinsic fatigue resistance, the study conducted by Merot et al. [51] will be examined. The researchers investigated the fatigue behavior of a 316L steel produced using laser powder bed fusion, which contained various populations of defects, including lack of fusion (LoF), corrosion pits (CP), and electric discharge machined hemispherical defects (EDM). The study found that the crack leading to failure consistently initiated from a single surface defect, and interestingly, the nature and morphology of the critical defect did not appear to have any influence on the fatigue strength. Instead, only the size of the defect seemed to matter. To incorporate the critical defect size into the analysis, the Murakami–Endo, El Haddad, and Chapetti models were implemented and calibrated. Subsequently, a modified Paris propagation law was employed to model the regime of short cracks and predict the Δσ-N curve domains based on the range of critical defect sizes.

The focus of this analysis lies in examining various concepts related to the application of fracture mechanics models for estimating the threshold curve, specifically in cases where inherent material defects resulting from processing are present. However, the comparison and potential advantages of different models will not be discussed in detail in this context. For a more comprehensive understanding of the topic, further insights can be gained by referring to references [11,44].

**Figure 7:**
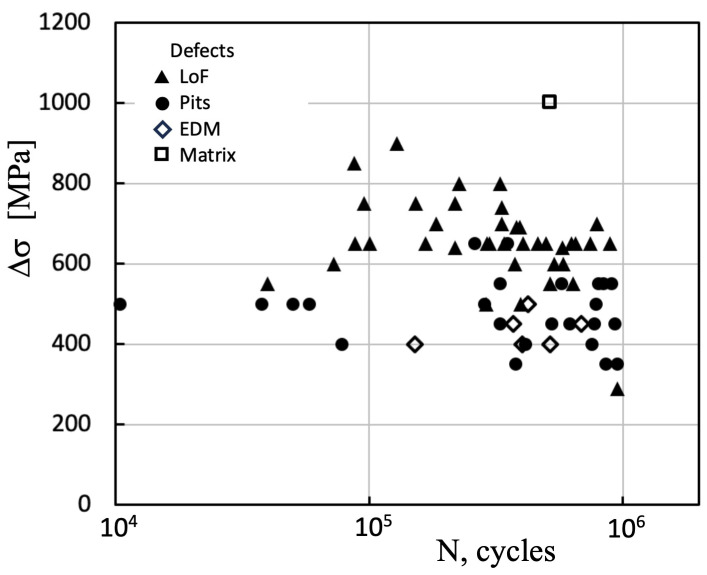
Fatigue life data was reported by Merot et al. for various populations of defects: lack of fusion (LoF), corrosion pits (Pits), and electric discharge machined hemispherical defects (EDM). The fatigue life for a failure from the matrix is also shown. *R* = −1 [51,52].

The observed results are influenced by the combined effect of the material matrix’s strength and the presence of inherent defects, which impact the damage process and the determination of fatigue resistance. Data reported by Merot et al. are: tensile strength σ_U_ = 642 MPa, Vickers hardness Hv = 225 Kgf/mm^2^, grain size *d* = 49 μm, and threshold for long cracks (load ratio *R* = −1) ΔK_thR_ = 9.04 MPa m^1/2^ [51]. Figure 7 illustrates the fatigue lives of all the tested specimens, with an indication of the type of defect leading to fracture [51,52]. It is important to note that the fatigue life reported by Merot et al. for failure initiated within the matrix (without involvement of defects) does not exceed 5 × 10^5^ cycles. Therefore, this value should not be considered representative of the intrinsic fatigue limit. From Figure 7, it is evident that if one were to extrapolate towards 10^7^ cycle lives using slopes similar to those observed in the remaining data, the intrinsic resistance would be significantly lower. However, Merot adopts an intrinsic fatigue limit Δσ_eR_ = 900 MPa based on the stress range level applied for the failure from the matrix and the data reported by Andreau for a similar material [53].

A similar analysis can be conducted concerning the fatigue lives of all other reported experiments. Notably, none of these fatigue lives exceed a million cycles, and more than half of them do not surpass 5 × 10^5^ cycles. These findings indicate that, in these cases, the applied stress intensity factor (ΔK) is considerably higher than the propagation threshold (ΔK_thR_) associated with the matrix. Lifetimes approaching 10 million cycles are expected to be associated with an applied ΔK close to the propagation threshold (ΔK_thR_) for crack growth. However, Merot et al. deviated from the conventional approach by modifying a parameter in each model using an adjustment derived from the collected data. This approach holds significant implications while drawing conclusions because it no longer estimates the propagation thresholds of the matrix (as the models typically do). Instead, the models are adjusted to fit the experimental results.

On the other hand, Merot et al. did not measure the propagation threshold for long cracks, ΔK_thR_. Instead, they reported a value of ΔK_thR_ equal to 9.04 MPa m^1/2^, which was obtained using the Murakami–Endo model with the parameter *area*^1/2^ equal to 300 μm and under the assumption that the range of short cracks had already been surpassed, indicating the threshold for long cracks had been reached.

Regarding the microstructural size, they estimated a value of *d* = 11.4 μm, which was obtained by using it as a fitting parameter when applying the Chapetti model to explain the experimental results. However, it is essential to note that they also measured the average microstructural size for the analyzed material, which was reported to be equal to 49 μm. In the current analysis, the measured microstructural side *d* = 49 μm is used in accordance with the assumptions of the Chapetti model.

To apply the Chapetti model to the experimental data reported by Merot et al., it is supposed that the Murakami–Endo and Chapetti models predict similar ΔK_dR_ values, as shown in Figure 6. The minimum ΔK_th_ (ΔK_dR_) is estimated using the Murakami–Endo model (Equation (5)) for *R* = −1, given ΔK_dR_ = 4.49 MPa·m^1/2^ for *d* = 49 μm. These values are associated with an intrinsic fatigue limit Δσ_eR_ = 497 MPa (according to Equation (4). When comparing this value with the tensile strength (642 MPa), it appears that the estimation made here is more reasonable than the value reported by Merot et al. (900 MPa).

Furthermore, to determine ΔK_thR_, the original Murakami–Endo model (Equation (5)) is employed instead of the modified model utilized by Merot et al. The parameter *area*^1/2^ is set to 300 μm, aligning with the assumption made by Merot et al. that the range of short cracks has already been surpassed, indicating the threshold for long cracks has been reached. The estimation yields a value of ΔK_thR_ = 7.62 MPa·m^1/2^.

**Figure 8:**
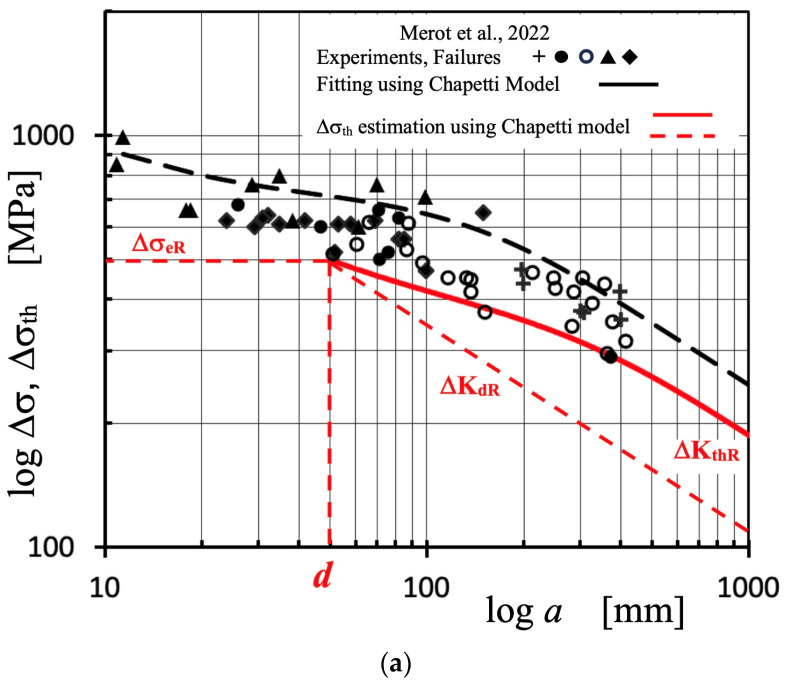

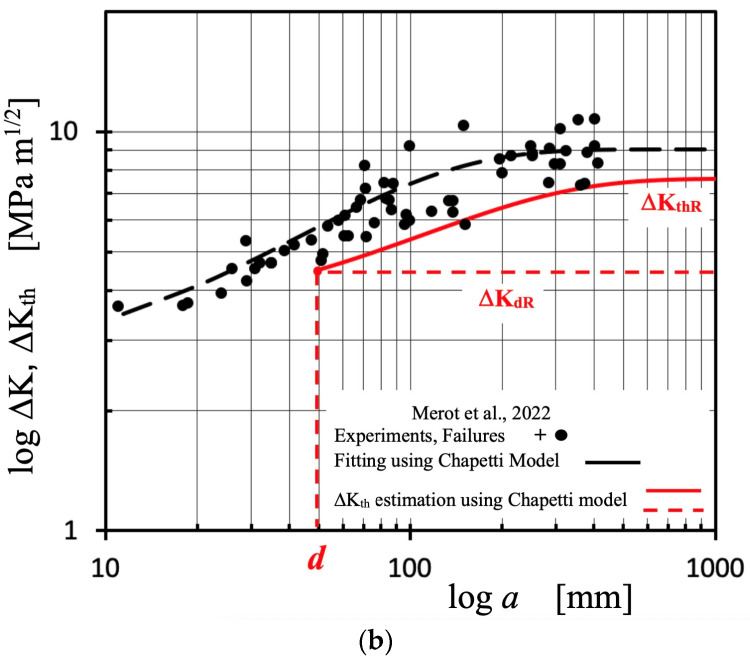
Data reported by Merot et al. (black symbols) for 316L steel and *R* = −1 [51,52], their threshold curve estimated using the Chapetti model and a fitting procedure (black dashed line), and the estimation using the Chapetti model developed here (red line). (**a**) Applied Δσ and threshold Δσ_th_ as a function of crack length (or defect size), K-T diagram. (**b**) Applied ΔK and threshold ΔK_th_.

In Figure 8, the data presented by Merot et al. is depicted using black symbols, along with their estimated threshold curve obtained using the Chapetti model and a fitting procedure (black dashed line). Additionally, the estimation developed here using the Chapetti model is represented by red lines. The crack length serves as the input variable, calculated by equating the parameter *area*^1/2^ to 1.253 times the crack length. This calculation assumes that the area corresponds to that of a semicircular surface crack. Figure 8a displays the Kitagawa–Takahashi diagram, while Figure 8b presents the same results but in terms of the stress intensity factor range.

As expected, nearly all of the experimental data lies significantly above the threshold curve, which represents the ΔK_th_ value associated with the fatigue limit as a function of defect size. The primary distinction between the two implementations of the Chapetti model lies in the assumptions, criteria, and methodologies employed to estimate the intrinsic fatigue limit, Δσ_eR_.

Additionally, there is an inherent uncertainty associated with the long crack threshold. It is important to bear in mind that when utilizing the Murakami–Endo model to estimate it, it is necessary to consider the upper limit of its validity, precisely determined by that threshold. The use of crack lengths exceeding the upper limit of the range of short cracks (*a*_sc_) may result in overestimations of the threshold, as illustrated in Figure 6b. If this were the case, the correct result would be a threshold curve with lower stress ranges for similar crack lengths, resulting in a better explanation of the analyzed fractures. These uncertainties highlight the need to experimentally measure or properly and conservatively estimate the threshold for long cracks, which is the upper limit value for the threshold curve.

## 4. ΔK_dR_ Estimation

Let us now analyze two formulas proposed here to estimate the intrinsic fatigue limit, Δσ_eR_, or alternatively, the associated propagation threshold, ΔK_dR_.

If Figure 6b is observed, it can be inferred that it would be possible to estimate the intrinsic strength of the material using the Murakami–Endo model (Equation (5)) and the microstructural size *d*, in the following manner (as it was proposed in [11]):

(7) ΔKdR=0.00356 HV+120 d13

where *d* is in μm and *H_V_* in kgf/mm^2^, given ΔK_dR_ in MPa.m^1/2^. In Equation (7), the relationship between *area*^1/2^ and the microstructural size *d* has been considered, assuming a semicircular crack of depth *d* (hypothesis of the Chapetti model).

**Figure 9:**
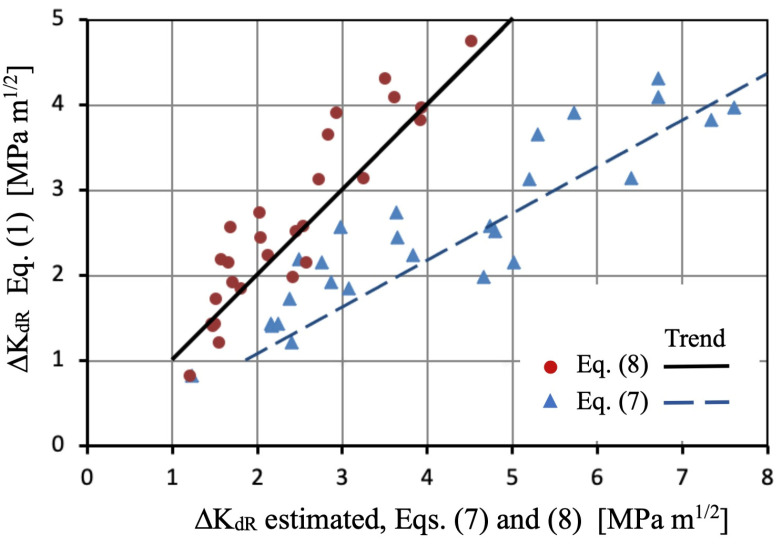
Comparison between ΔK_dR_ values given by Equation (1) for experimental data [22,24,29,55,56] and estimations using Equations (7) and (8).

On the other hand, Chapetti has proposed the following expression to estimate ΔK_dR_ for steels as a function of *H_V_* hardness and the microstructural size *d* [54]:

(8) $$\Delta K_{\mathrm{dR}}=1+0.5\;H_{V}\;\sqrt{\;\pi \;d\;}$$

where *d* is in m and *H_V_* in kgf/mm^2^, given ΔK_dR_ in MPa·m^1/2^. Figure 9 shows the ΔK_dR_ values given by Equation (1), along with the corresponding estimations obtained using Equations (7) and (8) for the steels data reported in [22,24,29,55,56]. The data utilized and the resulting estimations are presented in Table 2. The black line in Figure 9 represents the equality between the values obtained from Equation (1) and the estimations, indicating the satisfactory performance of Equation (8).

The results clearly demonstrate a higher estimation when using Equation (7), which assumes that the Murakami model allows estimating ΔK_dR_ for *a* = *d* (see Figure 6a). The overestimation of Equation (7) compared to Equation (8) remains at approximately 90%, yielding values almost twice as high. For a conservative estimation, Equation (8) could be preferred over Equation (7), although data collection and statistical analysis for different load ratios should be further explored in order to expand and improve it, even for other metallic alloys. To perform this task, it is necessary to gather sets of values for Δσ_eR_, *d*, and Hv of the analyzed materials.

## 5. Concluding Remarks

In some manufacturing processes, such as additive manufacturing, inherent defects can occur, which eliminate or minimize the initiation stage of fatigue cracks. This, combined with the advancements in understanding the behavior of small cracks and the development of fracture mechanics models that can predict it, has made the damage tolerance methodology the best tool for designing safe mechanical components. However, fracture mechanics analysis requires estimating the resistance curve of the material in the absence of defects. This estimation relies on knowledge of the intrinsic fatigue limit of the material, which represents its resistance without any defects.

This study aims to enhance our understanding of high-cycle fatigue damage and fracture mechanics models by exploring their fundamental concepts and assumptions. It introduces the concept of intrinsic fatigue resistance and provides experimental evidence that establishes its correlation with non-propagating cracks and the position of microstructural barriers. Furthermore, the study conducts comparative analyses of the Murakami–Endo, Chapetti, and crack closure-based models, uncovering disparities and limitations within these approaches. By incorporating intrinsic fatigue resistance, a recent publication showcased notable outcome variations, emphasizing its crucial significance in comprehending fracture mechanics models’ application.

From the analysis conducted and the results obtained, the following points can be emphasized:

- It is necessary to remember, consider, and explore in future work the hypothesis that the fatigue limit represents a threshold for the propagation of micro-cracks associated with the microstructural configuration (phase sizes, hardness, etc.) where they are generated.
- This hypothesis generates a minimum crack length value that represents the lower limit of validity for fracture mechanics models using the concept of a resistance curve (propagation threshold as a function of crack length) to estimate fatigue limits or high-cycle fatigue lives of metallic components.
- This minimum crack length value, defined in this study by the average microstructural size *d*, is, in turn, associated with a microstructural threshold in terms of the stress intensity factor range, ΔK_dR_, which should coincide with the estimated minimum propagation threshold predicted using the models. In the case of models based on the concept of crack closure, there is still a lack of overwhelming experimental evidence regarding this minimum value and its connection to the intrinsic fatigue limit.
- It is necessary to emphasize the limitations associated with the application of threshold prediction models for fitting procedures to experimental data that do not represent threshold configurations (and with associated fatigue lives lower than those considered run-outs for fatigue or endurance limits). For nominal applied stress range values higher than the fatigue limit of the analyzed material, fracture mechanics models should only be used for estimating finite fatigue lives, comparing the applied ΔK curves with the threshold ΔK_th_ curve.
- Two methods are proposed and compared for estimating the minimum threshold for crack propagation, ΔK_dR_, associated with the intrinsic fatigue limit. These methods are applied to experimental results reported in the literature. The first method (Equation (7)) involves determining the minimum threshold using the Murakami–Endo model for a crack length equal to the average microstructural size *d*. The second method (Equation (8)) utilizes a simple expression that also incorporates the Vickers hardness and the parameter *d* and provides lower estimation values that exhibit very good agreement with the experimental data for steels.
- The concept of intrinsic fatigue limit is then introduced and applied to reanalyze a recent publication, which has been very useful in clarifying the topics discussed.

## Figures and Tables

**Table 1:** *a*_0,eff_ and *d* comparison.

Material	*d* [μm]	*R*	Δσ_eR_ [MPa]	ΔK_th,eff_ [MPa m^1/2^]	*a*_0,eff_ Equation (1) [μm]	*a*_0,eff_/*d*	ΔK_dR_ [MPa m^1/2^]
Ti6Al4V [39]	20	−1	~900	1.9	4	~1/4	4.6
		0.1	450		13	~1/2	2.3
		0.4	340		23	~1	1.75
		0.6	250		44	~2	1.28
S20C [21]	55	−1	326	~2.6 *	48	~1	2.78
S20C [21]	7.8	−1	470		23	~3	1.51
		0	~235		92	~12	0.75
JIS SUJ2 [40]	10	−1	2400		~1	~1/10	8.7
SM41B [41]	14	−1	396		14	~2	1.7
2.25Cr1Mo [42]	25	−1	500		20	~1	2.9
		0	~250		81	~3	1.44
S10C [27]	55	−1	300		56	~1	2.56
S10C-CuP [27]	55	−1	600		14	~4	5.12
UFGS [29]	0.8	−1	800		8	~10	0.82

* Estimated with ΔK_th,eff_ ≈ 1.3 × 10^−5^ E [3,43], and E = 200 GPa.

**Table 2:** Steel data and ΔK_dR_ estimations.

Steel	*d* [μm]	Δσ_eR_ [MPa]	H_V_ [Kg/mm^2^]	ΔK_dR_ Equation (1) [MPa m^1/2^]	ΔK_dR_ Equation (8) [MPa m^1/2^] Chapetti	ΔK_dR_ Equation (7) [MPa m^1/2^] Chapetti-Murakami–Endo
S10C [55]	15.9	470	445	2.16	2.57	4.90
	30	495	355	3.12	2.72	3.80
	50	530	399	4.32	3.50	6.70
	89.9	350	347	3.82	3.91	7.30
S20C [55]	12.8	480	444	1.98	2.41	4.60
	27.4	520	483	3.14	3.24	6.30
	49.5	490	470	3.97	3.93	7.60
	80.4	460	442	4.75	4.51	8.50
SNC21 [55]	12	630	472	2.51	2.45	4.70
	24.9	680	437	3.91	2.93	5.70
S15C [56]	21	364	175	1.92	1.71	2.85
	43	362	175	2.73	2.02	3.6
	116	330	273	4.09	3.61	6.70
S35C [56]	8	442	185	1.44	1.46	2.13
	26	382	247	2.24	2.12	3.80
	67	388	252	3.66	2.83	5.30
S55C [56]	5	548	239	1.41	1.47	2.14
	6	510	228	1.44	1.49	2.20
	30	388	213	2.45	2.03	3.60
	6	432	254	1.22	1.55	2.38
S45C [22]	14	430	241	1.85	1.80	3.00
S10C [24]	55	300	103	2.56	1.68	1.65
UFG [29]	0.8	800	250	0.82	1.20	1.22
CGS [29]	12.5	550	187	2.24	1.57	2.48
